# Evaluation of the Course of Acute-Phase Reactants in the Postoperative Period of Newborns and Their Diagnostic Utility in Identifying Postoperative Sepsis

**DOI:** 10.3390/diagnostics16040545

**Published:** 2026-02-12

**Authors:** Erkan Deniz, Leyla Sero, Duygu Tuncel, Nilufer Okur

**Affiliations:** 1 Clinics of Pediatrics, Gazi Yasargil Training and Research Hospital, 21090 Diyarbakır, Turkey; 2 Clinics of Neonatology, Gazi Yasargil Training and Research Hospital, 21090 Diyarbakır, Turkey

**Keywords:** neonatal sepsis, procalcitonin, c-reactive protein, postoperative infection, biomarkers

## Abstract

**Background/Objectives**: Neonatal sepsis remains a major diagnostic challenge, particularly in postoperative infants where systemic inflammatory responses may mimic infection; therefore, reliable biomarkers are urgently needed to distinguish sepsis from normal post-surgical changes. **Methods**: This prospective cohort study included 135 neonates who underwent surgery without preoperative signs of infection. Serum levels of C-reactive protein (CRP) and procalcitonin (PCT) were measured preoperatively and at 24, 72 and 120 h postoperatively. Patients were classified as having proven sepsis based on positive blood cultures or clinical sepsis using European Medicines Agency (EMA) neonatal sepsis scoring. Biomarker levels were compared between septic and non-septic infants, and diagnostic performance was evaluated using receiver operating characteristic analysis. **Results**: Sixteen infants had proven sepsis and twenty-five had clinical sepsis. Both CRP and PCT levels showed a significant rise in septic infants at 72 h postoperatively (*p* < 0.01) and remained elevated at 120 h. PCT demonstrated the highest diagnostic accuracy at 72 h (AUC 0.911; threshold 1.2 µg/L; sensitivity 83%; specificity 84%), while CRP also showed good performance (AUC 0.802–0.838). **Conclusions**: CRP, PCT and albumin are valuable postoperative sepsis markers; however, single-parameter interpretation is insufficient, and a multiparametric, time-dependent approach provides superior diagnostic reliability in neonatal sepsis.

## 1. Introduction

The neonatal period is a critical stage characterized by the ongoing maturation of physiological, immunological, and metabolic systems, which renders infants highly vulnerable to external insults. Surgical interventions during this period impose significant physiological stress and trigger inflammatory processes that stimulate the immature immune system, thereby complicating systemic responses. Postoperative systemic inflammation and sepsis secondary to infection remain leading causes of morbidity and mortality, particularly among preterm and low birth weight neonates [1,2]. Clinical signs are variable and nonspecific, and the diagnosis and management of neonatal sepsis continue to present major challenges [3].

A positive microbiological blood culture is considered the gold standard for the diagnosis of neonatal sepsis. However, in cases of low-level bacteremia—which may account for up to two-thirds of neonatal sepsis cases—larger blood volumes are required to achieve reliable culture results [4]. Moreover, the delayed turnaround time of 2–3 days renders blood cultures unsuitable for the early diagnosis of sepsis [5].

Acute phase reactants (APRs) are plasma proteins synthesized primarily by hepatocytes in response to inflammation or tissue injury, showing rapid changes in serum levels. Triggered by proinflammatory cytokines such as interleukin-6 (IL-6), tumor necrosis factor-alpha (TNF-α), and interleukin-1β, these biomarkers may provide valuable information for differentiating infectious from noninfectious inflammatory processes [6,7]. In the postoperative setting, neonates are exposed to surgical trauma, tissue injury, and systemic inflammatory responses, which may mimic or overlap with infection. This diagnostic uncertainty often delays decisions regarding the initiation or discontinuation of antibiotics. Reliable markers capable of distinguishing infection from sterile inflammation in the postoperative period are therefore of critical importance.

C-reactive protein (CRP) and procalcitonin (PCT) are widely used biomarkers in clinical practice; however, their postoperative kinetics and correlation with infection have not been fully elucidated in the neonatal population [8]. CRP, synthesized in the liver in response to IL-6 and other cytokines, typically begins to rise 10–12 h after infection or tissue injury [9]. PCT, which is produced mainly by monocytes and hepatocytes in response to inflammatory cytokines, appears to be more specific to bacterial infections. Several studies have suggested that PCT may rise earlier and decline more rapidly than CRP [10]. Thus, serial measurements of APRs—particularly CRP and PCT—may provide a diagnostic advantage in distinguishing sepsis from postoperative inflammatory responses in neonates.

The aim of this study is to evaluate the predictive value of CRP and PCT levels for the diagnosis of sepsis in neonates during the postoperative period, to determine their sensitivity and specificity, and to identify potential threshold values for early prediction.

## 2. Materials and Methods

This prospective cohort study was conducted in a tertiary neonatal intensive care unit (NICU) between 12 February 2025 and 15 September 2025. Ethical approval was obtained from the local clinical research ethics committee (7 February 2025-830). All procedures were conducted in accordance with the ethical standards of the institutional and/or national research committee and with the principles outlined in the Declaration of Helsinki. Written informed consent was obtained from the parents and/or legal guardians of all participants prior to their inclusion in the study.

The role of postoperative CRP and PCT levels in predicting sepsis was investigated in neonates who underwent surgery in the NICU for various reasons. In our clinic, the routine surgical prophylaxis protocol consists of administering cefazolin (Cezol^®^, Abdi İbrahim Pharmaceuticals, Istanbul, Türkiye) at a dose of 30 mg/kg/dose (maximum 2 g) in two doses: 30 min before surgery and 12 h after surgery.

### 2.1. Inclusion Criteria

Infants with a corrected gestational age ≤ 44 weeks, who had not received antibiotics for proven or clinical sepsis/septic shock within 5 days prior to surgery, had no evidence of microbial growth in blood, cerebrospinal fluid, catheter, peritoneal fluid, or pleural fluid samples before surgery, and whose parents provided signed informed consent were included in the study.

### 2.2. Exclusion Criteria

Infants who had received preoperative antibiotic therapy for proven or clinical sepsis/septic shock, those with preoperative morbidities causing inflammation such as necrotizing enterocolitis, patients with life-threatening unstable hemodynamic status, those with incomplete or insufficient clinical/laboratory data, and cases where the parents declined participation or did not provide informed consent were excluded.

### 2.3. Study Protocol

In neonates who underwent surgery, blood samples were obtained at approximately 24, 72, and 120 h postoperatively (±6 h) for complete blood count, CRP (mg/L), PCT (µg/L), blood culture, blood gas analysis, and biochemical parameters.

- Proven sepsis was defined as the presence of clinical and laboratory findings consistent with sepsis, in which the causative microorganism was identified by blood culture or, in culture-negative cases, by molecular methods (Polymerase Chain Reaction, 16S rRNA), antigen-based assays, or other specific diagnostic techniques.
- Clinical sepsis was defined as cases with clinical and laboratory findings suggestive of sepsis but without microbiological confirmation in cultures. Patients with European Medicines Agency (EMA) neonatal sepsis scores ≥ 6 were considered as having clinical sepsis. EMA scoring provides a standardized “common language” for case definition; because it integrates clinical signs with laboratory findings, it was considered appropriate for use in this study [11].

Postoperative acute-phase reactants were compared between infants with proven or clinical sepsis and those without sepsis (No sepsis group). Patients who underwent abdominal surgery were compared with those who underwent non-abdominal surgery in terms of postoperative changes in CRP and PCT levels.

### 2.4. Demographic and Clinical Data

The following data were collected: birth weight (g), gestational age (weeks), sex, maternal characteristics (preeclampsia, HELLP syndrome, diabetes, premature rupture of membranes, oligohydramnios, polyhydramnios, chorioamnionitis, drug exposure), delivery characteristics (mode of delivery—cesarean section or normal spontaneous vaginal delivery—, need for resuscitation at birth, 5-min APGAR score), requirement for respiratory support, central catheter use, postoperative duration of mechanical ventilation, length of NICU stay, and postoperative clinical complications.

### 2.5. Laboratory Data

Complete blood count parameters [leukocyte count (µL), neutrophil count (µL), lymphocyte count (µL), hemoglobin (g/dL), platelet count (µL)], mean platelet volume (MPV, fL), C-reactive protein (CRP, mg/L), procalcitonin (PCT, µg/L), blood culture results, serum albumin level (mg/dL), and lactate level (mmol/L) were recorded. Antibiotic treatments administered to the patients were also documented.

### 2.6. Statistical Analysis

Data analysis was performed using SPSS for Windows, version 21.0. Descriptive statistics are presented as mean ± standard deviation or median (interquartile range, IQR) for continuous variables and as frequency (*n*) and percentage (%) for categorical variables.

- Normality testing: Data distribution was assessed with the Shapiro–Wilk test.
- Group comparisons: Independent samples t-test was used for parametric variables, and the Mann-Whitney U test was applied for nonparametric variables.
- Categorical variables: Chi-square test or Fisher’s exact test was used as appropriate.
- Multivariable logistic regression analysis was performed to evaluate the association between postoperative biomarker levels and sepsis status, adjusting for surgical type, birth weight, and APGAR scores.
- Diagnostic test performance: Receiver Operating Characteristic (ROC) curve analysis was performed to evaluate the predictive value of CRP and PCT levels for sepsis. Sensitivity, specificity, area under the curve (AUC), and optimal cut-off values were calculated. The Youden index was used to determine optimal cut-off thresholds.

A *p*-value < 0.05 was considered statistically significant for all tests.

## 3. Results

A total of 135 neonates were included in the study. Sixteen infants (11.8%) were classified as having proven sepsis, 25 (18.5%) as clinical sepsis, and 94 had no evidence of sepsis. The mean gestational age was 36 ± 3.4 weeks, and the mean birth weight was 2650 ± 754 g. Surgical procedures included myelomeningocele repair in 32 infants (23.7%), ventriculoperitoneal shunt placement for hydrocephalus in 26 (19.2%), esophageal atresia repair in 9, congenital diaphragmatic hernia repair in 4, gastrointestinal surgeries in 54, and other operations in 10 cases.

In the blood cultures of 16 patients with proven sepsis, two gram-negative microorganisms (*Enterobacter cloacae* and *Klebsiella pneumoniae*) were isolated, while coagulase-negative staphylococci were identified in 14 patients.

### 3.1. Demographic and Clinical Characteristics

Table 1 summarizes the demographic and clinical features of neonates with and without sepsis. Birth weight was significantly higher in the non-sepsis group (*p* = 0.027). The 5-min Apgar score was significantly lower in the clinical sepsis group (*p* = 0.010).

Central venous catheters (CVCs), predominantly umbilical venous catheters, were present in 51 patients (54%) in the no sepsis group, 13 patients (52%) in the clinical sepsis group, and 10 patients (62.5%) in the proven sepsis group. There was no statistically significant difference in the presence of CVCs between the groups (no sepsis vs. clinical sepsis, *p* = 0.424; no sepsis vs. proven sepsis, *p* = 0.376).

### 3.2. Laboratory Findings

Table 2 compares laboratory parameters between infants with clinical sepsis and those without sepsis. At postoperative 72 h, CRP (*p* = 0.04) and PCT (*p* < 0.01) levels were significantly higher in the clinical sepsis group. At 120 h, platelet count (*p* = 0.019) and albumin level (*p* = 0.008) were significantly higher in the non-sepsis group, whereas CRP (*p* = 0.002) and PCT (*p* < 0.01) remained significantly elevated in the clinical sepsis group.

When patients with culture-proven sepsis (*n* = 16) were compared with the remaining patients (clinical sepsis and no sepsis, *n* = 119), there were no significant differences in postoperative CRP and PCT levels at 24, 72, and 120 h (all *p* > 0.05).

In Table 3, the distributions of C-reactive protein and procalcitonin levels at postoperative 24, 72, and 120 h were compared between patients with proven sepsis (*n* = 16) and no sepsis (*n* = 94). While both C-reactive protein and procalcitonin levels were significantly higher in the proven sepsis group at postoperative 72 and 120 h, no statistically significant difference was observed between the groups at postoperative 24 h.

Abdominal surgery patients (*n* = 54) and those who underwent non-abdominal surgery (*n* = 81) were compared in terms of postoperative CRP and procalcitonin (PCT) levels at 24, 72, and 120 h using the Mann–Whitney U test. At postoperative 24 h, no significant difference was observed between the groups with respect to CRP levels (*p* = 0.892), whereas PCT levels were significantly higher in infants who underwent abdominal surgery (*p* < 0.001). At postoperative 72 h, CRP levels were similar between the two groups (*p* = 0.486); however, PCT levels remained significantly higher in the abdominal surgery group (*p* = 0.001). Likewise, at postoperative 120 h, there was no significant difference in CRP levels between the groups (*p* = 0.940), while PCT levels were significantly higher in infants undergoing abdominal surgery compared with those undergoing non-abdominal surgery (*p* = 0.007).

### 3.3. ROC Analysis

**Figure 1:**
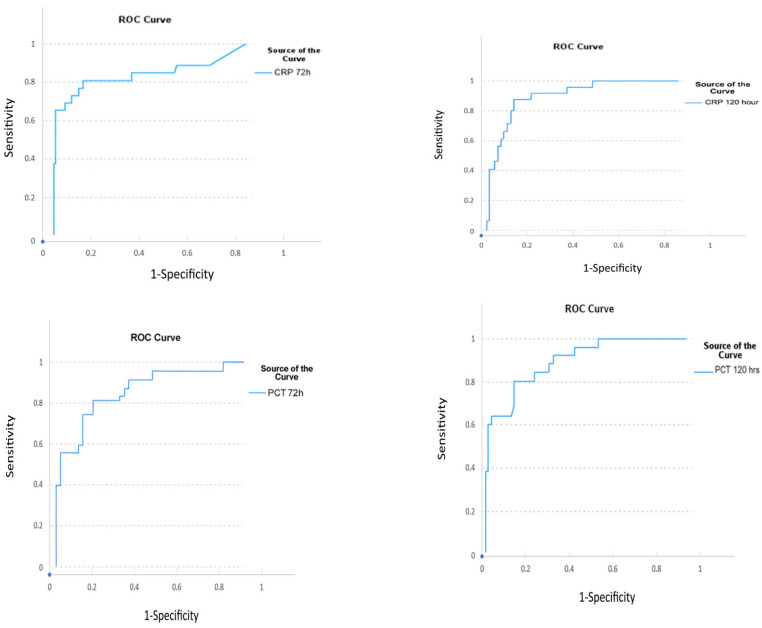
ROC analysis of CRP and PCT at postoperative 72 and 120 h.

Figure 1 and Table 4 present the results of ROC analysis. At 72 h postoperatively, PCT (AUC = 0.911, *p* < 0.01) and CRP (AUC = 0.802, *p* < 0.01) showed significant predictive performance. At 120 h, both PCT (AUC = 0.856, *p* < 0.01) and CRP (AUC = 0.838, *p* < 0.01) demonstrated high sensitivity and specificity in diagnosing clinical sepsis.

The ROC curves highlight the discriminative power of these biomarkers in sepsis diagnosis. At 72 h, PCT was the strongest marker (AUC = 0.911, *p* < 0.01, cut-off = 1.2 µg/L, sensitivity 83%, specificity 84%). CRP was also significant (AUC = 0.802, *p* < 0.01, cut-off = 20 mg/dL, sensitivity 79%, specificity 79%). At 120 h, PCT (AUC = 0.856, *p* < 0.01, cut-off = 0.57 µg/L, sensitivity 78%, specificity 83%) and CRP (AUC = 0.838, *p* < 0.01, cut-off = 10.13 mg/dL, sensitivity 78%, specificity 77%) both remained significant. These results indicate that both CRP and PCT are useful in sepsis diagnosis, with PCT demonstrating superior diagnostic performance, particularly at 72 h.

### 3.4. Postoperative Kinetics of Biomarkers

**Figure 2:**
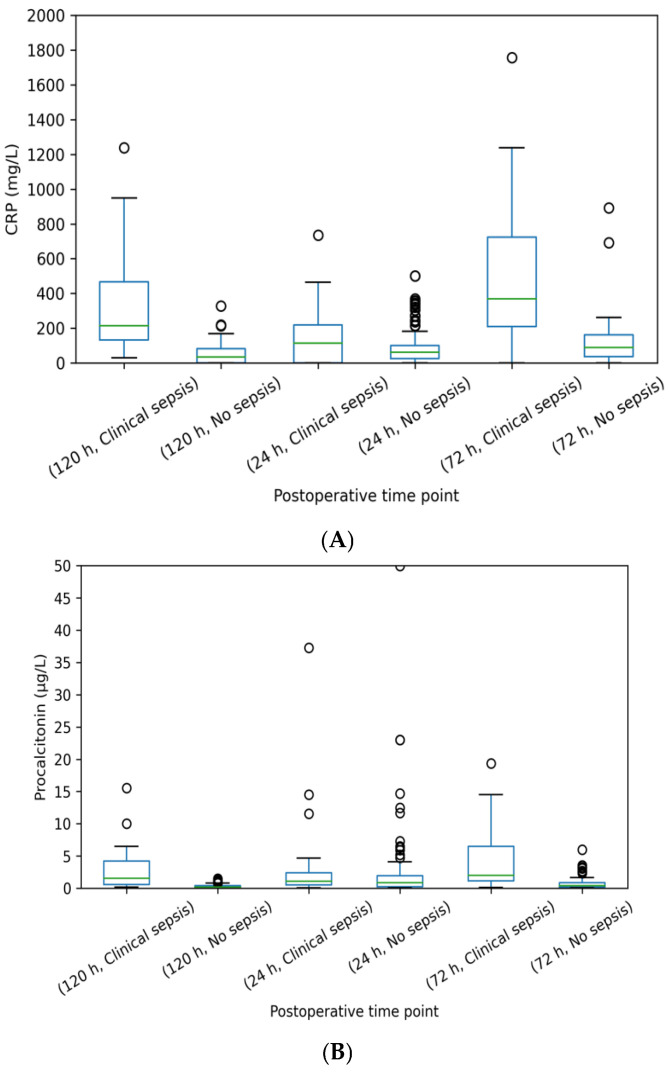
(**A**) Graph of PCT values at postoperative 24, 72, and 120 h in patients with clinical sepsis and no sepsis. (**B**) Graph of CRP values at postoperative 24, 72, and 120 h in patients with clinical sepsis and no sepsis.

- Figure 2A. Boxplots illustrating postoperative serum CRP levels (mg/L) at 24, 72, and 120 h in neonates with clinical sepsis and without sepsis. Boxes represent the median and interquartile range (IQR), whiskers indicate the range of non-outlier values, and individual dots denote outliers. The boxplot presentation allows clear visualization of data distribution and relative differences between groups across postoperative time points.
- Figure 2B. Boxplots illustrating postoperative serum PCT levels (µg/L) at 24, 72, and 120 h in neonates with clinical sepsis and without sepsis. Data are displayed according to sepsis status and time point using uniform axis scaling. Boxes represent the median and interquartile range (IQR), whiskers indicate the range of non-outlier values, and dots represent outliers. This visualization highlights the temporal divergence of PCT levels between septic and non-septic infants, particularly at 72 h after surgery

Multivariable regression analysis was performed, and the independent associations between postoperative biomarkers and clinical and culture-proven sepsis were evaluated. For clinical sepsis, no significant associations were found between CRP and PCT levels at 24 h (*p* > 0.05); however, higher birth weight was associated with a reduced risk of clinical sepsis (*p* = 0.013), and the 5-min APGAR score was inversely associated with clinical sepsis (*p* = 0.043). At postoperative 72 and 120 h, CRP and PCT levels were identified as independent and significant predictors of clinical sepsis (72 h: CRP *p* = 0.018, PCT *p* < 0.001; 120 h: CRP *p* = 0.007, PCT *p* = 0.006), and abdominal surgery showed a significant inverse association with clinical sepsis during these periods (*p* = 0.006 and *p* = 0.027, respectively). For culture-proven sepsis, no significant associations were observed with biomarkers at 24 and 120 h (*p* > 0.05); only CRP levels at 72 h were independently and significantly associated with culture-proven sepsis (*p* = 0.049), whereas PCT levels did not reach statistical significance and showed only borderline significance (*p* = 0.063) (Table 5).

## 4. Discussion

In this single-center cohort, we demonstrated the temporal dynamics of CRP and PCT levels after non-cardiac surgery in neonates and evaluated the performance of predictive thresholds for sepsis. Among 135 neonates undergoing surgery, the incidence of clinical sepsis was 18.5% and proven sepsis 11.8%. At 72 and 120 h, both PCT and CRP levels were significantly higher in the clinical sepsis group; ROC analysis revealed that PCT at 72 h had the highest discriminatory power (AUC = 0.911), outperforming CRP. This result aligns with recent systematic reviews and meta-analyses, which consistently show superior diagnostic accuracy of PCT compared with CRP in neonatal sepsis. A large meta-analysis published in The Lancet Child & Adolescent Health confirmed that PCT provides significantly better diagnostic performance and achieves a more favorable sensitivity–specificity balance in the early course of infection [12]. Similarly, systematic reviews and meta-analyses published in 2023 and 2024 concluded that while both PCT and CRP individually demonstrate “good” discriminatory ability, PCT offers greater support for clinical decision-making, particularly within the first 72–120 h [13].

In our study, optimal cut-off values were 1.2 µg/L for PCT and 20 mg/dL for CRP at 72 h, while lower thresholds at 120 h (0.57 µg/L for PCT and 10.13 mg/L for CRP) yielded high discriminatory performance. These time-dependent shifts in thresholds reflect biomarker kinetics and the resolution of postoperative sterile inflammation. Reported cut-off values for neonatal sepsis vary between 0.5–2.0 ng/mL for PCT; thresholds above 0.5 ng/mL increase sensitivity but show marked variability in specificity across centers and populations [14]. Moreover, postoperative inflammatory responses can cause a more sustained rise in CRP, whereas PCT is considered more specific to bacterial triggers and less influenced by sterile inflammation after 48–72 h. This concept has been repeatedly emphasized in pediatric cardiac surgery studies [15] and may explain the superior ROC performance of PCT at 72 h in our cohort.

A clinically relevant finding was the absence of significant differences in CRP and PCT levels within the subgroup of culture-proven sepsis. Potential explanations include limited statistical power due to the small number of culture-positive cases, reduced sensitivity of blood cultures following early initiation of antibiotics, technical limitations related to restricted blood volumes, and possible misclassification of true infections as “clinical sepsis” when cultures remained negative despite biomarker elevation. Current evidence suggests that the variable sensitivity of cultures in neonatal sepsis underscores the importance of integrating biomarker-guided decision-making, ideally through serial measurements combined with clinical indices [13]. Supporting this, a prospective study in 2024 reported that bedside point-of-care measurement of PCT, CRP, and IL-6 accelerated clinical decision-making and optimized antibiotic stewardship [9].

There is no strong or consistent evidence that biomarker kinetics in neonatal sepsis fundamentally differ between culture-positive and culture-negative cases. Studies demonstrate substantial overlap between the two groups; although culture-positive sepsis often shows higher peak biomarker levels and more prolonged elevations, these differences are strongly influenced by factors such as sampling time, gestational age, and infectious burden. The delayed rise and limited early discriminatory value of C-reactive protein, along with the inability of procalcitonin to reliably distinguish culture-positive sepsis when used alone, further support this overlap. While early-response biomarkers such as interleukin-8 and presepsin may better discriminate culture-positive cases in some studies, overall biomarker kinetics appear to reflect the severity of the inflammatory response rather than culture status itself. Therefore, biomarkers should be interpreted independently of culture results and always in conjunction with serial measurements and the clinical context [16,17,18].

Lower birth weight in the non-sepsis group and lower 5-min Apgar scores in the clinical sepsis group are consistent with known perinatal risk patterns for impaired neonatal defense capacity. Multiple studies support associations between birth weight, perinatal depression markers, and sepsis risk. However, data remain limited regarding how these variables modulate biomarker thresholds, and the development of center-specific algorithms is recommended [13].

The thresholds we obtained are clinically applicable. At 72 h, a PCT cut-off of 1.2 µg/L yielded 83% sensitivity and 84% specificity. Compared with the widely reported 0.5 ng/mL threshold, which emphasizes sensitivity, this higher cut-off optimizes specificity and may be more suitable for NICUs with high prevalence of clinical sepsis and significant postoperative inflammatory burden. In line with this, Roa et al. prospectively studied 60 neonates undergoing major non-cardiac surgery and found significantly higher CRP and PCT levels at 72 and 96 h in the definite/probable sepsis group versus non-sepsis, with optimal cut-off values at 96 h of 74.16 mg/L for CRP and 1.65 ng/mL for PCT. Combined positivity of both markers achieved 100% specificity, whereas positivity of either increased sensitivity to 88.9% [19]. Our findings parallel these results, showing high sensitivity and specificity for both PCT and CRP at 72 and 120 h. The lack of significant differences in proven sepsis cases in our study likely reflects the limitations of blood culture yield.

Neunhoeffer et al. investigated IL-6, PCT, and CRP in the first two postoperative days and reported that all increased significantly after surgery, with higher levels in sepsis compared to SIRS. IL-6 peaked immediately after surgery, CRP at 24–48 h, and PCT at 24 h. IL-6 demonstrated the highest early sensitivity, while CRP and PCT provided stronger discrimination in the subsequent days [20]. Although IL-6 was not assessed in our cohort, both PCT and CRP showed high sensitivity and specificity for predicting sepsis at postoperative days 3 and 5. Studies in older pediatric populations have produced mixed results. For example, in 22 pediatric liver transplant patients (aged 19 days to 16 years), PCT had poor diagnostic accuracy (AUC = 0.52), while CRP performed better (AUC = 0.89) [21]. In children after cardiac surgery, retrospective data indicated no significant differences in CRP levels between infected and non-infected groups, whereas PCT levels became significantly higher after day 4 in infected patients [22].

In neonatal sepsis, both CRP and PCT increase in the presence of bacterial infection; however, the biomarker profiles associated with Gram-negative (GN) and Gram-positive (GP) pathogens are not consistently reported across studies. In culture-confirmed bacteremia/sepsis cohorts, PCT levels—particularly within the first 72 h—have been shown to be higher in GN infections than in GP infections, suggesting superior discriminatory performance for predicting GN pathogens. In contrast, findings regarding CRP are more heterogeneous: while some studies report no significant differences in CRP levels between GN and GP infections, more recent data indicate that CRP and PCT concentrations may diverge according to pathogen type in neonatal sepsis, and that combined interpretation of these biomarkers may enhance diagnostic discrimination [23,24,25]. In our cohort, coagulase-negative staphylococci accounted for 87.5% of culture-positive isolates; therefore, no confounding pattern in acute-phase reactants was observed.

In addition to overall agreement with existing literature, certain divergences must be noted. Some centers report that CRP alone may provide acceptable accuracy, and under specific thresholds, its diagnostic value approaches that of PCT. Nevertheless, at the systematic review and meta-analysis level, PCT consistently maintains superiority [26]. It should also be acknowledged that PCT can transiently rise after surgery without infection, and over-interpretation outside the clinical context may increase inappropriate antibiotic use, a risk quantitatively demonstrated in pediatric cardiac cohorts [15]. Therefore, biomarkers should always be interpreted in conjunction with clinical findings, hemodynamic indices, and, when available, secondary markers such as IL-6, nCD64, or presepsin. Emerging evidence suggests that combined biomarker panels may further improve diagnostic accuracy [27].

In neonatal sepsis caused by viral infections, acute-phase reactants show a distinct pattern. CRP usually remains within normal limits or increases only mildly and nonspecifically, often reflecting tissue injury or non-infectious inflammation rather than true infection. In contrast, PCT typically does not rise in viral infections, as interferon-γ suppresses its synthesis, resulting in lower levels compared with bacterial sepsis. Therefore, CRP has limited discriminatory value in viral infections, whereas persistently low PCT levels provide more reliable differentiation between viral and bacterial sepsis and may help guide antibiotic decision-making [28].

Abdominal procedures are traditionally considered to increase the risk of postoperative infection due to intestinal manipulation and potential bacterial translocation. Therefore, the inverse association observed in our study is more likely attributable to case-mix confounding rather than a true protective effect. In our cohort, neonates undergoing abdominal surgery were generally more clinically stable, underwent planned procedures, and received standardized perioperative antibiotic prophylaxis with close postoperative monitoring. Consequently, abdominal surgery should be interpreted as a surrogate marker of a specific patient subgroup rather than an independent determinant of reduced sepsis risk, and this finding should not be considered causal.

Strengths of our study include standardized serial sampling at fixed time points (24, 72, 120 h) and the concurrent ROC analysis of both PCT and CRP, which allowed us to propose clinically practical cut-offs. Limitations include the single-center design, small sample size in the proven sepsis subgroup, heterogeneity of surgical procedures, and potential confounders such as corticosteroid exposure, renal function, and transfusions—all of which are known to independently influence biomarker kinetics and complicate cut-off interpretation. In addition, the EMA sepsis score represents one of the limitations of our study. In a multicenter prospective cohort study, the diagnostic performance of the EMA neonatal sepsis criteria for identifying proven neonatal sepsis was found to be limited. In that study, the sensitivity, specificity, and overall accuracy of the EMA score were reported as 44.2%, 64.4%, and 55.1%, respectively. Owing to the relatively low specificity, the false-positive rate was approximately 35.6%, indicating that nearly half of EMA-positive cases (around 48%) did not have culture-proven sepsis. The EMA sepsis score has the potential to overclassify sterile inflammatory responses occurring in the postoperative period as clinical sepsis. Postoperative findings such as surgical stress–related systemic inflammatory response syndrome, hemodynamic instability, respiratory distress, and elevations in inflammatory markers may lead to EMA positivity in the absence of infection. [29]. In this context, it should be acknowledged that the EMA sepsis score may have overestimated the rate of clinical sepsis in postoperative neonates.

## 5. Conclusions

CRP and PCT are valuable biomarkers for distinguishing sepsis from postoperative inflammation in neonates. In this prospective cohort, PCT demonstrated superior diagnostic accuracy compared with CRP, particularly at 72 h after surgery, with clinically applicable cut-off values. Serial monitoring of these biomarkers may support earlier diagnosis, optimize antibiotic use, and improve clinical decision-making in postoperative neonatal care.

## Figures and Tables

**Table 1:** Demographic and Clinical Characteristics of the Study Groups.

	Proven Sepsis (*n* = 16)	Clinical Sepsis (*n* = 25)	No Sepsis (*n* = 94)	*p*
Gestational age, weeks *	36.1 ± 4.2	35.9 ± 3.7	36.9 ± 1.7	0.148
Birthweight, g *	2712 ± 870	2558 ± 850	2855 ± 530	0.027
Male, *n* (%)	7 (44)	12 (48)	43 (45.7)	0.901
C/S, *n* (%)	6 (37.5)	10 (40)	46 (49)	0.901
Apgar score at 5th min **	8 (6–9)	7 (6–9)	8 (5–10)	0.010
Postnatal age at surgery, day **	4 (1–86)	4 (1–86)	3 (1–36)	0.452
Duration of postoperative invasiv Mechanical ventilation, day **	0 (0–4)	0 (0–27)	0 (0–39)	0.696
Mortality, *n* (%)	1 (6.3)	2 (8)	7 (7.4)	0.245

* Mean ± Standard Deviation. ** Median (min–max).

**Table 2:** Comparison of acute phase reactants and selected laboratory findings between patients with clinical sepsis and no sepsis.

	Clinical Sepsis (*n* = 25)	No Sepsis (*n* = 94)	*p*
Preoperative			
WBC (×1000/µL) *	12.8 ± 4.7	15 ± 4.3	0.25
Neutrophil (×1000/µL) *	6.7 ± 3.5	7 ± 4.1	0.86
Lymphocyte (×1000/µL) *	7 ± 3.8	5.5 ± 2.6	0.76
Platelet (×1000/µL) *	238 ± 91	310 ± 126	0.37
MPV, fL *	9.7 ± 0.9	10 ± 1	0.12
Albumin g/dL	31 ± 5.3	31 ± 4.1	0.507
Lactate mmol/L *	3.1 ± 1.4	2.8 ± 1.2	0.298
C-reactive protein, mg/L **	0 (0–9.6)	0 (0–8.6)	0.509
Procalcitonin, µg/L **	0.17 (0.07–3.5)	0.16 (0–4.6)	0.5
Postoperative 24th hour			
WBC (×1000/µL) *	12 ± 7.2	12.4 ± 4.1	0.93
Neutrophil (×1000/µL) *	8.3 ± 5.3	7.6 ± 3.7	0.77
Lymphocyte (×1000/µL) *	2.4 ± 1.9	3.3 ± 1.6	0.05
Platelet (×1000/µL) *	248 ± 194	296 ± 142	0.75
MPV, fL *	10.3 ± 1.2	10 ± 1	0.27
Albumin g/dL	23.5 ± 3.4	25 ± 4.1	0.056
Lactate mmol/L *	2.6 ± 1.5	2.5 ± 1.2	0.84
C-reactive protein, mg/L **	11.5 (0–80.7)	0 (0.09–50)	0.146
Procalcitonin, µg/L **	1.09 (0.08–241)	0.96 (0.06–148)	0.518
Postoperative 72nd hour			
WBC (×1000/µL) *	11.5 ± 5.6	12 ± 4.5	0.310
Neutrophil (×1000/µL) *	7.5 ± 5	6.6 ± 3.7	0.429
Lymphocyte (×1000/µL) *	3.3 ± 1.9	3.8 ± 1.7	0.14
Platelet (×1000/µL) *	287 ± 185	346 ± 163	0.125
MPV, fL *	10.6 ± 1.2	10.3 ± 1.2	0.289
Albumin g/dL	22 ± 4.2	25 ± 3.9	0.054
Lactate mmol/L *	3.1 ± 1.4	2.8 ± 1.2	0.489
C-reactive protein, mg/L **	26.2 (0–175)	11.5 (0–223)	0.04
Procalcitonin, µg/L **	1.6 (1–183)	0.45 (0.06–148)	<0.01
Postoperative 120th hour			
WBC (×1000/µL) *	12 ± 7	12.6 ± 5	0.660
Neutrophil (×1000/µL) *	7.1 ± 5.3	5.3 ± 3.4	0.174
Lymphocyte (×1000/µL) *	3.9 ± 1.9	4.6 ± 1.9	0.103
Platelet (×1000/µL) *	327 ± 173	432 ± 199	0.019
MPV, fL *	10.6 ± 1	10 ± 1.2	0.353
Albumin g/dL	23.5 ± 4.4	26.5 ± 5	0.008
C-reactive protein, mg/L **	13.3 (0–76.6)	4.9 (0–124)	0.002
Lactate mmol/L *	2.7 ± 1.4	2 ± 0.8	0.152
Procalcitonin, µg/L **	1.99 (0.16–140)	0.2 (0.05–10)	<0.01

* Mean ± Standard deviation. ** Median (Minimum-Maximum). WBC: White blood cell, MPV: Mean Platelet Volume.

**Table 3:** Comparison of acute phase reactants and selected laboratory findings between patients with proven sepsis and no sepsis.

	Proven Sepsis (*n* = 16)	No Sepsis (*n* = 94)	*p*
**Postoperative 24th hour**			
C-reactive protein, mg/L *	15.5 (0–73.5)	6.1 (0–50)	0.093
Procalcitonin, µg/L *	1.09 (0–37.3)	0.09(0.06–148)	0.436
**Postoperative 72nd hour**			
C-reactive protein, mg/L *	25 (0–175)	10.6 (0–89)	0.026
Procalcitonin, µg/L *	1.4 (0.1–14.5)	0.43 (0.07–6)	0.04
**Postoperative 120th hour**			
C-reactive protein, mg/L *	10.2 (0–76.6)	3.5 (0–33)	0.013
Procalcitonin, µg/L *	0.47 (0.08–15.6)	0.19 (0.05–1.52)	0.043

* Median (Minimum-Maximum).

**Table 4:** Cut-off values, sensitivity, and specificity of CRP and PCT levels at postoperative 72 and 120 h for clinical sepsis.

	AUC	*p*	Cut-Off Value	Sensitivity (%)	Specificity (%)	Asymptotic 95% Confidence Interval	
						Lower Bound	Upper Bound
CRP 72 h (mg/L)	0.802	<0.01	20	79	79	0.686	0.924
PCT 72 h (µg/L)	0.911	<0.01	1.2	83	84	0.853	0.971
CRP 120 h (mg/L)	0.838	<0.01	10.13	78	77	0.764	0.939
PCT 120 h (µg/L)	0.856	<0.01	0.57	78	83	0.789	0.943

CRP: C-Reactive Protein, PCT: Procalcitonin, AUC: Area Under the Curve.

**Table 5:** Multivariable Logistic Regression Analysis for Clinical and Proven Sepsis.

	Odds Ratio (Exp(B))	95% Confidence Interval	*p* Value
*Clinical sepsis*			
Birth weight, g	0.90	0.83–0.97	0.013
Apgar score at 5 min	0.86	0.75–0.99	0.043
CRP at 72 h	1.05	1.01–1.10	0.018
PCT at 72 h	1.62	1.30–2.02	<0.001
Abdominal surgery	0.42	0.23–0.76	0.006
CRP at 120 h	1.07	1.02–1.13	0.007
PCT at 120 h	2.85	1.35–6.01	0.006
Abdominal surgery	0.55	0.33–0.92	0.027
*Proven sepsis*			
CRP at 72 h	1017	0.994–1.040	0.043

## Data Availability

The original contributions presented in this study are included in the article. Further inquiries can be directed to the corresponding author.

## Abbreviations

The following abbreviations are used in this manuscript:

AUC	area under the curve
CRP	c-reactive protein
IL-6	interleukin-6
MPV	mean platelet volume
NICU	neonatal intensive care unit
PCT	procalcitonin
TNF-α	tumor necrosis factor-alpha
WBC	white blood cell
